# Improved brace design combining CAD/CAM and finite element simulation for the conservative treatment of adolescent idiopathic scoliosis (AIS): preliminary results of a randomized control trial

**DOI:** 10.1186/1748-7161-10-S1-O59

**Published:** 2015-01-19

**Authors:** Carl-Éric Aubin, Nikita Cobetto, Julien Clin, Frederique Desbiens-Blais, Hubert Labelle, Sylvie Le May, Stefan Parent

**Affiliations:** 1 Polytechnique Montreal, Montreal, Canada; 2 Research Center, Sainte-Justine University Hospital Center, Montreal, Canada; 3 Université de Montréal, Montreal, Canada

## Objectives

In a previous study on 15 AIS patients, we demonstrated the feasibility of a brace design technology combining Computer Aided Design and Manufacturing (CAD/CAM) and finite element modeling (FEM) for the treatment of scoliosis. The braces showed an equivalent correction to standard braces, but were 61% thinner and had 32% less material. A Randomized Control Trial (RCT) was undertaken to pursue the validation and further assess the effectiveness of braces issued from this technology as compared to standard brace design approaches.

## Material and methods

Patients who received a brace treatment prescription were randomized into two groups: 1) TLSO (thoraco-lumbo-sacral orthosis) fabricated using a scan of patient's torso and a CAD/CAM approach (Rodin4D) (StandardBrace); 2) TLSO additionally designed using Finite Element Modeling (FEM) built from 3D reconstructions of the trunk skeleton from bi-planar radiographs and optimization (NewBrace). The latter approach allowed to simulate the scoliosis correction and the applied pressures on the torso, and to iteratively design the brace up until the simulated correction was considered maximal and its contact surface with the torso minimal. It was then fabricated using Computer Numerical Controlled technology. StandardBrace and NewBrace effectiveness was assessed using radiographs and compared to the simulations.

## Preliminary results

36 patients were consecutively enrolled and to date, 24 patients received their brace. The average Cobb angle prior to bracing was 32° and 30° for the main thoracic and lumbar curves respectively. The preliminary results showed that the NewBrace reduced Cobb angles by 47%, which was simulated with a difference inferior to 5°. The StdBrace reduced the Cobb angles by 40%. The NewBrace had 32% less covering surface than the StdBrace. The highest pressures were located on the thoracic and lumbar regions and at the axillary and the trochanter extensions.

## Preliminary conclusion

So far, a novel design scheme combining CAD/CAM and 3D FEM simulation allowed the fabrication of braces more efficient and lighter than standard TLSO. The study is ongoing to fully compare and validate the effectiveness of the novel design platform.

## Consent

Written informed consent was obtained from the patient for the image(s) used in this study. A copy of the written consent is available for review by the Editor of this journal.

**Figure 1:**
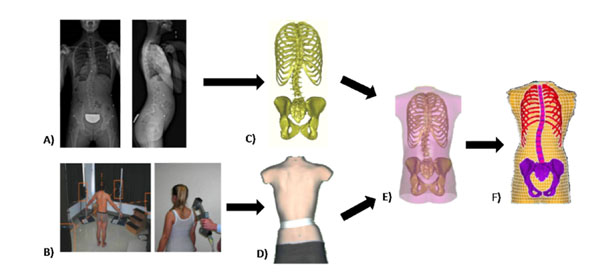
A) Acquisition of the internal geometry using the calibrated bi-planar radiographic 3D reconstruction technique; B) Acquisition of the external geometry using a surface topography system or a scan system; C) Internal 3D geometry; D) External 3D geometry E) Geometric registration F) Finite element model of the trunk

**Figure 2:**
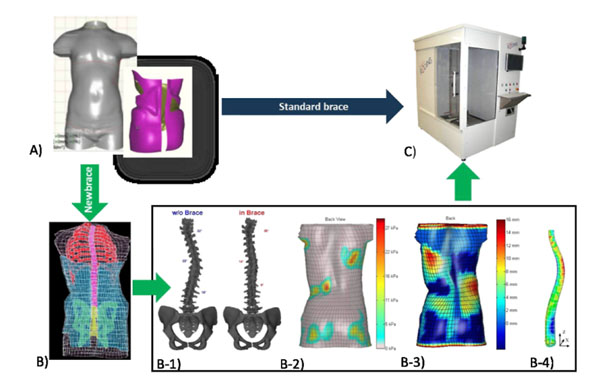
A) Brace design using the CAD software for Standard brace or Newbrace; B) Simulation of the brace installation; B1) Simulation of the spine correction; B2) Simulation of the applied pressures; B3) Simulation of the distance between the brace shell and the patient's skin (the blue color represents the material in contact with the patient's skin and the green, yellow, orange and red colors represent the brace material situated at more than 6 mm of the patient's skin); B4) Simulated pressures on epiphyseal growth plates; C) Brace fabrication using CAM milling system

**Figure 3:**
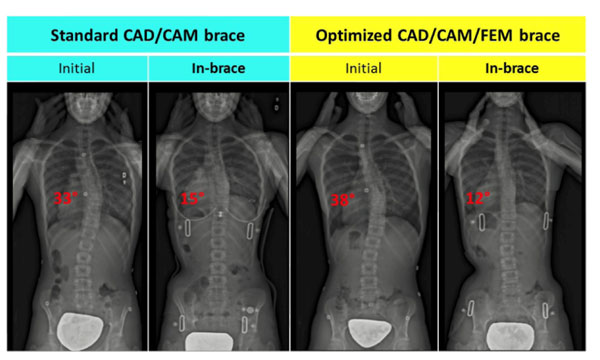
Radiographic results for two typical patients: out of brace for both patients (initial curve), with the StdBrace or with the NewBrace (depending of the randomization), in the postero-anterior view.

